# A rapid realist review of literature examining Co‐production in mental health services for youth

**DOI:** 10.1002/jcv2.12272

**Published:** 2024-08-21

**Authors:** Verity Rose Jones, Justin Waring, Nicola Wright, Sarah‐Jane Fenton

**Affiliations:** ^1^ University of Birmingham Birmingham UK; ^2^ University of Nottingham Birmingham UK

**Keywords:** adolescents, CAMHS, co‐production, mental health, participatory research, youth

## Abstract

**Background:**

An overview of internationally published literature on what works for co‐production in youth mental health services is missing, despite a practice and policy context strongly recommending this approach. This rapid realist review develops a theory about how and why co‐production methods in youth mental health services work, for whom and in which circumstances.

**Methods:**

Relevant evidence was synthesised to develop Context–Mechanism–Outcome configurations (CMOs) that can inform policy and practice. Stakeholders were iteratively involved by engaging an expert panel including young people and a separate youth advisory group. Searches across eight databases and expert panel suggestions identified 5716 documents which were screened at abstract level, 532 at full‐text and 57 documents were included in the review.

**Results:**

Data extracted from 57 papers contributed to five CMO configurations to describe the generative mechanisms by which co‐production in youth mental health services are linked to outcomes and influenced by context. The final programme theory is: Youth experts by experience (YEBE), particularly those from minoritised communities, provided with a supportive organisational culture can experience authentic engagement where their knowledge is perceived as credible by wider stakeholders. This leads to personal development for participating YEBE as well as service improvements from their input.

**Conclusions:**

Rich data from a heterogenous sample of papers along with stakeholder consultation enabled this review to refine a clear programme theory for co‐production in mental health services for young people. Nevertheless, further information is needed to identify what constitutes a supportive organisational culture and to explore rival theories or under‐evidenced areas.

## Graphical abstract

Relevant evidence was synthesised to develop context–mechanism–outcome (CMO) configurations about how and why co‐production methods in youth mental health services work, for whom and in which circumstances. These can inform policy and practice. Stakeholders were iteratively involved by engaging an expert panel including young people and a separate youth advisory group. Results: Key to effective co‐production in this context is a supportive organisational culture, sufficient resourcing, transparency about the limits for change and adult stakeholders sharing power with young people.

**Key Points**

**What's known?**

- Youth participation, including co‐production, is increasingly used and recommended in mental health service design and delivery to better understand and meet the needs of service users. We do not know for whom this works, in what circumstances and why.

**What's new?**

- Using a realist approach to interrogate literature on co‐production in mental health services for youth to identify what works.
- Key to effective co‐production in this context is a supportive organisational culture, sufficient resourcing, transparency about the limits for change and wider stakeholders sharing power with young people.

**What's relevant?**

- This paper offers practical learnings for those using co‐production with young people in clinical practice or service development. Areas requiring further research are identified.

## INTRODUCTION

The rise in mental ill‐health for young people across the world has been described as a global public health crisis (McGorry et al., 2022), with estimates that one in seven 10‐19‐year‐olds experience a mental disorder (WHO, 2021). There is broad consensus that mental health services for young people frequently fail to meet the needs of service users. Moreover, human rights abuses continue to occur within mental health services, and people frequently encounter harm when they seek help or are subject to compulsory treatment in these systems (Katterl et al., 2023; United Nations, 2023). The role of lived experience in shaping and delivering future services though participation work has been widely promoted as a possible route to system reform, to ensure people who use mental health services find these are appropriately configured to their needs (NDTi, 2016; Roper et al., 2018; Slay & Stephens, 2013). Different theorists have repeatedly conceptualised degrees of service‐user participation as ladders or continuums, dating back to Arnstein's (1969) ladder of citizen participation. Though there are differences in terminology, in most cases service users have little power or tokenistic participation at the bottom and at the top they are in control or share control (Heap et al., 2022). It is important to note that the definition of co‐production is contested (Jones et al., 2023).

Despite contested definitions, when co‐production features on these various scales, it is at or towards the top. Amongst these various degrees of lived experience participation, the principles and practices of co‐production have been proposed to have particularly transformative potential for addressing harms and facilitating service improvement and change (NICE, 2016; Robert et al., 2022; WHO, 2022b). There is also strong evidence that most long‐term mental ill health has its onset by adolescence (Department of Health and Social Care, 2021; McGorry et al., 2022), thus young people are a critical cohort when co‐producing mental health services. For this review the following definition of co‐production from the service user group Think Local Act Personal is used:

Co‐production is an equal relationship between people who use services and the people responsible for services. They work together, from design to delivery, sharing strategic decision‐making about policies as well as decisions about the best ways to deliver services. (Think Local Act Personal, 2021, p. 364)

Existing literature reviews in this area do not focus on youth, on co‐production specifically, on co‐production of services, or do not use a review method which takes a realist approach to identify ‘what works?’. Instead, they ask ‘does it work?’. An initial scope of the literature was completed to find existing reviews on co‐production in youth mental health services. Five were identified that consider adjacent topics (McCabe et al., 2023; Norton, 2021; Slay & Stephens, 2013; Viksveen, Erlend Bjønness, et al., 2022; Yamaguchi et al., 2022). The reviews from McCabe et al. (2023), Slay and Stephens (2013), Viksveen, Erlend Bjønness, et al. (2022) and Yamaguchi et al. (2022) do not focus solely on co‐production in youth mental health services. Yamaguchi et al.’s review (2022) is focussed on policymaking and Slay and Stephens’s (2013) review covers both adult and youth mental health together. Viksveen et al.’s systematic review (2022) covered user involvement more broadly. This included co‐production amongst other types of user participation or engagement, but it is isolating the specificity of co‐production that is of interest to this review. A recent systematic review of youth engagement in mental health research (McCabe et al., 2023) provides some insight into effectively co‐producing with this cohort, though these recommendations for using co‐production principles for conducting research may not be transferable to the context of co‐producing within services themselves. The fifth review (Norton, 2021) focussed specifically on co‐production in youth mental health, however included only two papers (38 participants) and highlighted the paucity of literature available.

Thus, despite these relevant reviews, the gap remains for a comprehensive overview of evidence specific to how co‐production works in youth mental health services. This review contributes to this gap by scrutinising papers describing co‐production that has that has been implemented into real‐world mental health practice or services for young people. Research questions are listed in Table 1.

**TABLE 1:** Research questions.

Overall Q	For whom and in what circumstances does co‐production work in youth mental health services?
Sub‐Q context	What are the important contextual factors in understanding co‐production in youth mental health services?
Sub‐Q mechanisms	What generative mechanisms explain the impact of co‐production in youth mental health services?
Sub‐Q outcomes	What are the outcomes for service users and services that result from co‐production in youth mental health services?

## MATERIALS & METHODS

The complex procedure associated with realist review can be difficult to navigate. This section contains the following subsections to maximise transparency and clarity in the methods followed:

- Changes in the Review Process from the Protocol
- Study Design: Why a Rapid Realist Review?
- Stakeholder Involvement in Developing the Research Question and Preliminary Programme Theory
- Search Strategy
- Document Selection and Appraisal
- Data Extraction, Analysis and Synthesis: Programme Theory Refinement and Validation

### Changes in the Review Process from the protocol

This review was designed according to the process outlined by Saul et al. (2013) for rapid realist reviews, prospectively registered with PROSPERO (CRD42023456623) and a protocol published (Jones et al., 2023). Realist And Meta‐narrative Evidence Syntheses: Evolving Standards (RAMESES) reporting standards (Wong et al., 2013) recommend identifying where changes are made from the method described in a protocol as best practice. Accordingly, these were as follows:

1. Forward and backward citation searching was not required as the search across eight databases provided sufficient data sources; 5716 documents were screened at abstract level, 532 at full‐text and 57 documents were included in the review.
2. A two‐stage screening process was adopted. For title and abstract screening, broad and inclusive criteria were used and consequently papers were deliberately over‐included at this stage. A second, more stringent, set of inclusion/exclusion criteria were utilised for full‐text screening (see Tables 2 and 3). This was because it was often difficult to ascertain whether the participation work described would count as co‐production or would sit on a lower rung of a participation hierarchy. Despite efforts to operationalise co‐production during protocol development (for example by excluding papers if young people were not included from the outset or including papers if equal power sharing was explicitly sought in the programme described), in practice during screening these decisions were not clear‐cut. The two‐stage screening approach addressed this and only documents found to describe generative mechanisms which contributed to programme theory during the relevance/richness/rigour assessment were included for data extraction as shown on Supporting Information S1: Figure S1.

### Study Design: Why a Rapid Realist Review?

This is the first realist review to synthesise evidence on co‐production in mental health services for youth. Using a rapid approach, which uses experts and stakeholders to support the searching and theory refinement, ensures that findings are relevant to service users, practitioners and policy makers and are responsive to the complex and changing contexts of mental health services. Though systematic reviews are widely used in healthcare research, such an approach is not appropriate in this instance because there is a paucity of evidence that could be included if assessed using a traditional evidence hierarchy tool, as was seen in a recent systematic review on the subject which included just two studies (Norton, 2021). Norton (2021) indicates that the search strategy in his paper will likely have excluded relevant studies and suggests a different review approach should be attempted for this topic. In response to this, a rapid realist review approach that includes grey literature, expert panel suggestions and realist quality appraisal was used here (Saul et al., 2013). Through this, many more relevant studies qualified for inclusion as they contained pertinent causal information that contributed to theory building.

Moving away from traditional evidence hierarchies, documents are instead screened for whether they can meaningfully contribute to the programme theory by assessing their relevance, richness and rigour. A rapid realist review is therefore most appropriate in this instance because this approach is inclusive of a wide range of literature and aims to provide practical guidance to practitioners and policymakers on how to alter the context or resources most likely to trigger generative mechanisms which produce the hoped‐for outcomes for complex programmes (Wong et al., 2013).

### Stakeholder Involvement in Developing the Research Question and Preliminary Programme Theory

**FIGURE 1:**
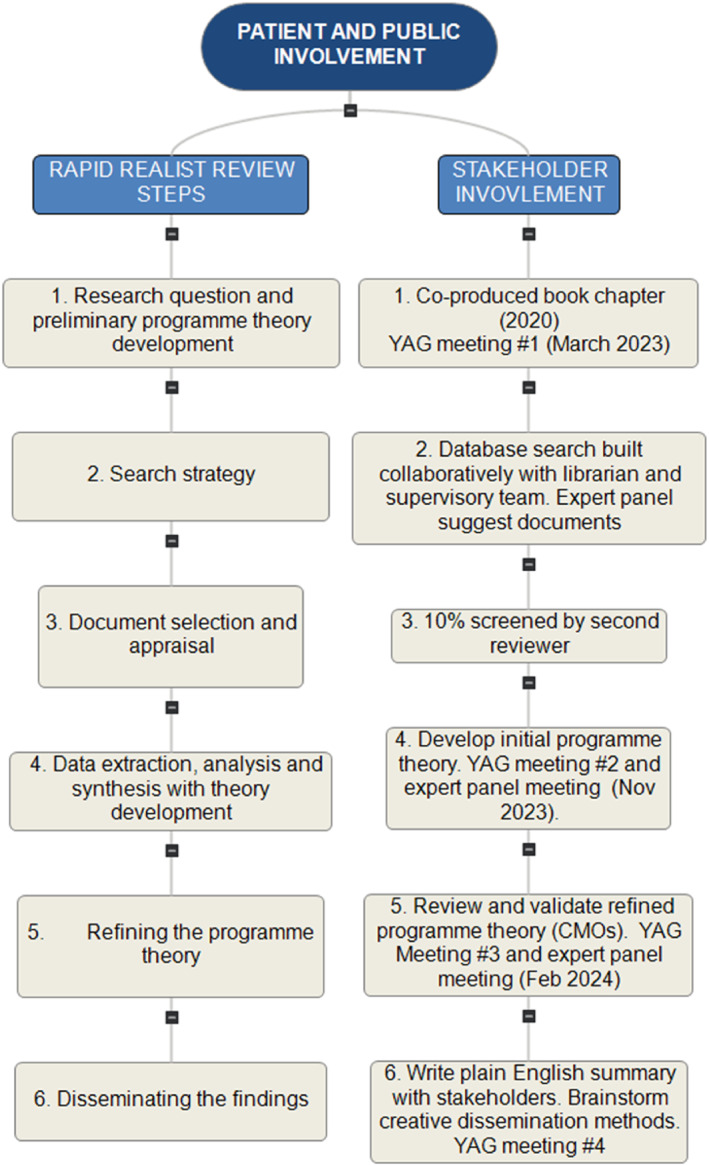
Stakeholder involvement throughout the stages of the rapid realist review.

In line with Saul et al.’s (2013) recommended steps for a rapid realist review the area of interest and research question were identified and refined with stakeholders. See Figure 1 for stakeholder involvement throughout the review. In 2020, the Institute of Mental Health's Youth Advisory Group at the University of Birmingham (hereafter referred to as the ‘youth reference group’) co‐wrote a book chapter on youth involvement in mental health service design and delivery in conjunction with researchers in the field (Fenton et al., 2020). The chapter considered the available literature and concluded that regarding youth involvement in mental health services, ‘We don't know what works, for whom, in what circumstances, and why’ (Fenton et al., 2020, p. 177), highlighting an area requiring further research to answer, and inspiring this review. Using this chapter to identify the review topic ensures that the review is focussed on a question that stakeholders are most interested in answering, because the chapter authors consisted of young people with lived experience of mental illness, lived experience researchers, and subject experts. Following this, the lead author met with the youth reference group in March 2023 to refine the research question (see Table 1). This meeting led to restructuring the research question to emphasise the ‘for whom’? component, as the reference group felt that identifying who is included and excluded and why is the area of research which is of utmost importance. Accordingly, the research question begins with this question, and the final programme theory centres ‘who’ in answering the question.

Preliminary programme theories were developed to support the search strategy through workshops with the youth reference group in March 2023 (five participants) and with researchers in the field in August 2023 (10 participants). These workshops are described in further detail in the review protocol (Jones et al., 2023). The priorities identified in both workshops, as well through initial scoping of relevant literature and feedback from the review expert panel were collated into a table listing possible contexts, generative mechanisms, and outcomes of interest. Preliminary programme theories (hypotheses) in the form of ‘if…then statements’ (a commonly used technique for realist theorising (Jagosh, 2023)) were produced from these by the lead author. The youth reference group (five participants) and expert panel (five participants) met in two online workshops in November 2023 to review these ‘if…then statements’ and these were edited during the workshops to reflect their feedback.

The expert panel comprised of a second group of young people with relevant lived experience embedded within a youth mental health service, a researcher with expertise in the field and a participation professional. Young people in both the reference group and expert panel were recruited based on experience of or a strong interest in youth mental health. These groups are embedded within a UK university and a UK statutory mental health service. Participants were renumerated in line with involvement guidelines (NIHR, 2022). The resulting initial programme theory is available in the supplementary material (Supporting Information S1: Table S2).

### Search Strategy

Three key concepts were identified for the literature search: mental health services, youth and co‐production. Synonym lists were generated from these concepts with input from the PhD supervisory team and a subject librarian. Full lists of the terms used are included as supplementary material in Supporting Information S1: Tables S3–10. Title, abstract and keyword searching was then completed for each synonym list, separated by ‘AND’ operators on eight databases: PsycINFO (Ovid), CINAHL plus (Ebsco), Medline (Ovid), Web of Science (Core Collection), SCOPUS, Social Policy and Practice (Ovid), ProQuest Dissertations & Theses Global, ASSIA (ProQuest). In addition, the expert panel members contributed suggestions of key papers. Covidence was utilised to remove duplicates and collate bibliographic records. Papers retrieved from both the database search and expert panel suggestions were screened against inclusion/exclusion criteria (see Tables 2 and 3).

**TABLE 2:** Inclusion and exclusion criteria for abstract and title screening.

Inclusion Criteria
Published in English
Focussed on young people (aged 10‐25)
Focussed on mental health
Describes lived experience participation work which may qualify as co‐production
Empirical studies and grey literature
Exclusion criteria
Literature reviews
Commentary without case study
Refers exclusively to shared‐decision making

**TABLE 3:** Inclusion and exclusion criteria for full‐text screening.

Inclusion Criteria
Papers which:
Described two‐directional participation between youth and service providers
Were concerned with real‐world co‐production of/in mental health services for youth (can be service‐initiated or research‐initiated)
Met the criteria for relevance, richness and rigour (see Supporting Information S1: figure S1)
Exclusion criteria
Papers which
Were concerned with one‐directional participation practices (e.g. informing, consulting)
Did not progress to real‐world implementation. The output is co‐designed but not implemented. (Examples of outputs: Research findings/interventions/apps/websites/service design)

### Document Selection and Appraisal

Titles and abstracts were screened, and full‐text versions sought if the source met the inclusion criteria. A second author (S‐JF) screened 10% of papers at the title and abstract screening stage. The authors met and discussed the sample and found agreement in how to apply the inclusion/exclusion criteria, using the two‐stage process described above. Literature in realist reviews is assessed for richness (is there enough detail?), scientific rigour (trustworthiness and coherence), and relevance to the research question (Dada et al., 2023; Saul et al., 2013; Wong et al., 2013), and these criteria were used to identify if a study contributes to a theory and/or testing theory and should therefore be included according to the process recommended by Dada et al. (2023) as shown on Supporting Information S1: Figure S1. The lead author assessed the selected papers for quality against these criteria and a sample of papers excluded based on this appraisal were checked by the three other authors (S‐JF, JW, NW). Full agreement was found across this sample. As suggested by Wong (2018), triangulation was used where scientific rigour was found to be low, in these cases additional sources of data were sought to support the contribution to the programme theory, or if this was unavailable then the data was excluded from theory development.

### Data extraction, analysis and synthesis: Programme Theory Refinement and Validation

Data extraction, analysis and synthesis, defined as step seven by Saul (2013), was broken down in this review into nine smaller steps to provide additional rigour, as described step‐by‐step in Table 4 below. The resulting refined programme theory is a middle‐range theory and thus intended to be applicable across co‐production initiatives for mental health services treating youth. The full extraction spreadsheets are available in the supplementary materials – table S15.

**TABLE 4:** Data extraction, analysis and synthesis.

*Steps*	Examples
**Step 1:** Data describing characteristics of the study/project were recorded in a MS Excel spreadsheet.	Supporting Information S1: Table S11: Study/Project characteristics data extraction
**Step 2:** Causal statements from the literature were extracted in a MS Excel spreadsheet for: context, mechanism – resource, mechanism – response and outcome. A summary statement was created for each of these extracted CMOs identifying the causal link between the mechanism resource and response.	Supporting Information S1: Table S12: Examples of CMO data extraction
**Step 3:** The complete set of causal summary statements were put onto virtual post‐it notes on a Miro whiteboard.	Supporting Information S1: Figure S2: Grouping by Demi‐Regularities
**Step 4:** Patterns in the data (known as ‘demi‐regularities’ (Jagosh, 2019)) were then grouped by theme on the Miro whiteboard.	Supporting Information S1: Figure S2: Grouping by Demi‐Regularities
**Step 5:** Each theme was summarised in a table recording the extracted contexts, mechanisms and outcomes contributing each theme.	Supporting Information S1: Table S13: Contexts, mechanisms and outcomes by Theme
**Step 6:** An ‘if…then’ statement was developed for each theme, and these were then used to construct CMO diagrams.	Supporting Information S1: Table S14: Theory refinement
**Step 7:** The diagrams were refined into five CMO configurations.	Results section below
**Step 8:** Connections between the CMO configurations were formulated into a conceptual map.	Supporting Information S1: Figure S3: Conceptual Map of full programme theory
**Step 9:** The refined programme theory was validated with stakeholders in online workshops to ensure findings from the literature reflect the experiences and learnings of practitioners and lived experience experts, and to identify any gaps.	

The preliminary programme theory in the form of ‘if…then statements’ developed with stakeholder groups was iteratively used to support the identification of CMOs during data extraction from the literature. Some CMOs which were not identified in the preliminary theory also emerged. The preliminary theory was therefore developed and refined into the five CMOs through the data extraction, analysis and synthesis in steps 2–7 below. Additionally, an important part of realist review is using a retroductive approach and for this review this involved incorporating middle‐range theories to confirm or refute the CMOs which emerged. Relevant substantive theories for each of the five CMOs are identified and discussed below in the results section. These were identified through consulting lists of middle‐range theory, consulting theory commonly applied in the subject area and through wider reading of the authors.

## RESULTS

### Document characteristics

**FIGURE 2:**
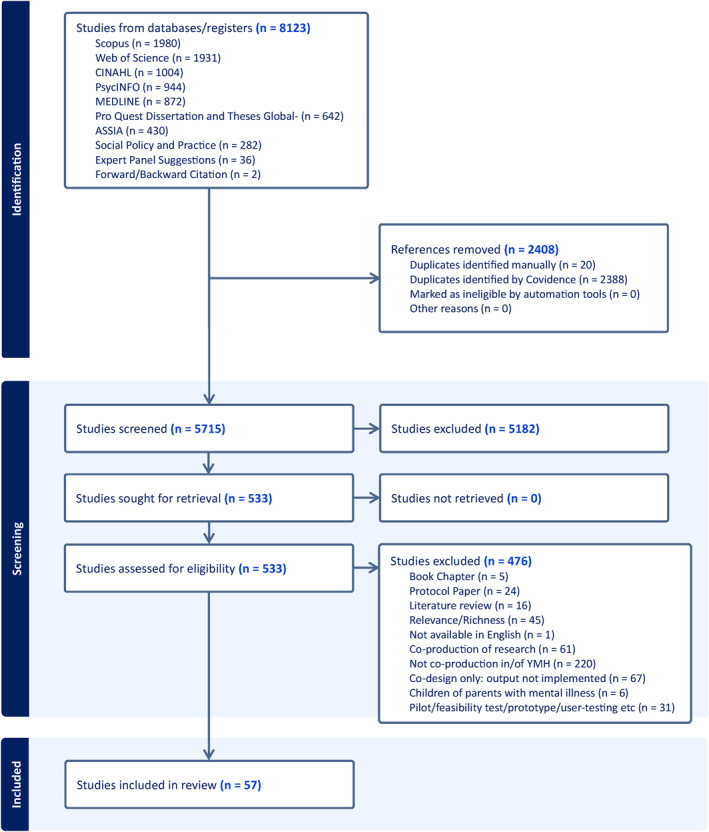
Search strategy and results.

In total, data extracted from 57 papers contributed to refining the initial programme theory and developing CMOs. Figure 2 shows the search strategy and results. Included papers were primarily from the global north (Australia 18, UK 16; Ireland 2; North America 17 [*Canada 9, USA 8*], New Zealand 1, Netherlands 1) with two from the global south (Indonesia 1, India 1). Across the 57 papers, 51 co‐production initiatives were described with 39 organisations represented. Mental health services described in the papers included statutory (health and local/state/national government), third sector (charitable, non‐governmental), private health services and research‐initiated programmes. Academic literature published in peer‐reviewed journals made up the majority of included papers (49). These comprised qualitative research (17); mixed methods research (9); quantitative research (1) and papers described here as ‘praxis’ (22). Praxis papers are journal articles which are not research but describe specific co‐production projects/programmes with young people in mental health service design/delivery which have been implemented into a practice or service setting. The remaining eight papers were grey literature including evaluation reports (4); a service leaflet (1); A briefing (1) and news articles from health professional publications (2).

### Methods of Co‐production Reported

**FIGURE 3:**
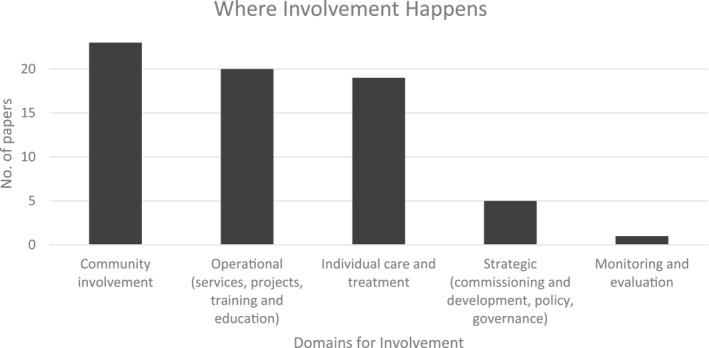
Where involvement happens (domains from the National Involvement Partnership Team (2014)).

Across the included papers (*n* = 31) the most common method of co‐production identified was young people employed in expert by experience roles (paid and voluntary). These roles included peer support workers, peer educators, youth leaders with public speaking roles and young people delivering staff training. Other common methods were youth advisory groups and young people co‐delivering services (see Supporting Information S1: Figure S4 for the full range of co‐production methods). The most common outputs from these were: prevention/promotion programmes and campaigns (involvement domain: community involvement), co‐delivery of treatment interventions (involvement domain: individual care and treatment) and service design (involvement domain: operational). Distribution across domains is shown in Figure 3. There were also papers reporting youth involvement in website/app design, strategy/evaluation/standards, discovery college courses, staff recruitment/training and three papers where it was unclear what the material output of the co‐production programme was (see Supporting Information S1: Figure S5 for the full range of co‐production outputs).

### Summary of findings and discussion in relation to substantive theory

A summary table of included papers numbered 1‐57 is available in the supplementary materials (Supporting Information S1: Table S16), corresponding numbers are used below to identify papers that contributed to each theory in Tables 5, 6, 7, 8, 9.

**TABLE 5:** Theory 1 CMO and evidence.

CMO configuration	Papers contributing
YEBE participants draw on their youth and lived experience (context) to improve services. When the organisational culture is supportive (mechanism – resource) YEBE's knowledge is perceived as credible by wider stakeholders (mechanism – response) leading to authentic engagement and increasing the likelihood the service will use their input to make improvements (outcome).	1, 2, 3, 5, 6, 9, 12, 15 16, 17, 22, 27, 28, 30, 32, 36, 38, 41, 42, 44, 45, 51, 56 (perceived as credible) 8, 13, 21, 27, 30, 43, 52 (not perceived as credible)

**TABLE 6:** Theory 2 CMO and evidence.

CMO configuration	Papers contributing
When YEBE participate in authentic co‐production (mechanism – resource) of mental health services (context) they benefit from personal development of 1. psychological recovery 2. New professional skills and 3. New knowledge (mechanism – response), they can use these personal gains to make changes more effectively to mental health services (outcome).	3, 8, 11, 13, 15, 23, 26, 29, 30, 32, 35, 38, 39, 40, 42, 43, 44, 46, 48, 56
*Rival theory: participating harms YEBE (e.g. through re‐traumatisation or disappointment)*	45

**TABLE 7:** Theory 3 CMO and evidence.

CMO configuration	Papers contributing
Structural or institutional systems around co‐production programmes often limit the scope for change (context), when services are transparent about these limits (mechanism – resource) the young people feel authentically respected and use this knowledge (mechanism – response) to plan achievable goals which are more likely to be implemented (outcome).	28, 42, 54 (clear expectations) 3, 11, 41 (lack of transparency)
*Rival theory: transparency leads to assimilation*	6

**TABLE 8:** Theory 4 CMO and evidence.

CMO configuration	Papers contributing
In mental health services for youth (context) where YEBE share decision‐making power and responsibility with wider stakeholders (mechanism – resource) relationships of mutual respect and trust are built where all parties feel listened to (mechanism – response) leading to services which are more responsive to the needs and preferences of all (outcome).	1, 2, 3, 6, 10, 13, 16, 18, 19, 20, 24, 26, 28, 31, 37, 43, 48, 49, 50, 53, 57 (*Shared power and responsibility)* 2, 3, 47, 49 (*Power and responsibility not shared*)

**TABLE 9:** Theory 5 CMO and evidence.

CMO configuration	Papers contributing
When a programme has sufficient resources and is flexible to new ways of working (mechanism‐resource) a more diverse group of young people who better represent the target group feel supported and valued (mechanism – response). Consequently, based on these YEBE's input the mental health service is more likely to offer culturally appropriate support (outcome) because YEBE from minoritized communities best understand their needs (context).	4, 7, 17, 23, 25, 32, 43, 45, 52, 55, 56 (sufficient resources and flexibility) 4, 7, 23, 34, 41, 43 (insufficient resources and inflexibility)

Theory 1 youth and lived experience → credible.

Theory 1 relates to whether youth lived experience and knowledge of mental ill health is perceived as credible. Evidence from 31 papers contributed to this CMO configuration (see Table 5). 24 papers linked credibility to service improvement and seven papers suggested that when Youth experts by experience (YEBE) knowledge is seen as not‐credible by wider stakeholders (e.g. mental health service staff) the impact of this form of youth participation on service improvement is limited. This is supported by the substantive theory of epistemic injustice, proposed by Fricker (2007). This is the theory that the knowledge held by some groups is prejudicially understood to be more legitimate than the knowledge of others. For co‐production in youth mental health services, this is likely to be where professional knowledge is considered superior to knowledge gained from lived experience of mental illness or distress. Additionally, for this cohort their youth can also mean their knowledge is doubted. Palmer et al. (2019) writes about epistemic injustice in the context of co‐production and co‐design, explaining that less powerful social groups are more likely to experience this. Thus, epistemic injustice can be further compounded for individuals from minoritised communities who hold less social power (e.g. those minoritised based on race/ethnicity, religion, sexuality, gender identity etc.) making it even more likely for those with intersecting characteristics (e.g. a young person of colour with lived experience of mental ill health) that their input be dismissed rather than perceived as credible.

The theory proposed In this CMO is therefore well supported by the papers from the review and the middle‐range theory of epistemic injustice. However, what remains less clear are definitions of the concepts within the CMO: what constitutes an organisational culture of support for, or resistance to, co‐production with YEBE?, how is organisation defined? What does this ‘authentic’ engagement look like for stakeholders? Supportive organisational culture is somewhat identified further in CMOs 3, 4 and 5 however throughout this review there was little within the literature that allowed the theory proposed in the CMOs to be tested against real‐world practice examples. Therefore, further stakeholder validation and exploration of how to cultivate and implement an organisational culture supportive for authentic youth co‐production would be beneficial.

Theory 2 participating → benefits/harms.

Theory 2 relates to whether participation is beneficial for YEBE. Data contributing to this theory strongly supported the mechanism‐response that individual young people benefit from participating in co‐production work, with 20 papers describing this (see Table 6). Psychological recovery was described variously including as improvements in: self‐esteem, self‐efficacy, confidence, feeling empowered, social connectedness and resilience (Davis‐Brown et al., 2012; Honig et al., 2006; Howe et al., 2011; Kenny et al., 2006; Le et al., 2022; Leijdesdorff et al., 2022; Lindstrom et al., 2021; Liza Hopkins, Foster, & Nikitin, 2018; Mayer & McKenzie, 2017; Norfolk and Suffolk NHS Foundation Trust, 2023; Oliver et al., 2006). New professional skills described included public speaking, communication, time management and teamwork (Crofts et al., 2017; Howe et al., 2011; Leijdesdorff et al., 2022; Norfolk and Suffolk NHS Foundation Trust, 2023; Oliver et al., 2006). Whilst new knowledge included knowledge of the mental health system, research and evaluation processes, and mental health conditions (Davis‐Brown et al., 2012; Howe et al., 2011; Mehrotra et al., 2017; Young et al., 2023).

Substantive theory supporting this can be found across the literature of occupational therapy and occupational science in which practitioners use activity/occupation that is meaningful to a client as the therapeutic medium of intervention to support psychological recovery and skills development (College of Occupational Therapists, 2006; Law et al., 2002). It is understood by occupational therapists that all people have both a ‘need and a right to engage in meaningful occupations throughout their lives’(American Occupational Therapy Association, 2011, p. 65) and that such occupation is intrinsically therapeutic. This substantive theory lends support to the identified mechanism‐response that YEBE participating in co‐production work benefit psychologically and vocationally from this occupational participation.

However, there was minimal data to support the outcome of this CMO that this personal benefit leads to material change within services. One paper theorised that young people participating can build connections with ‘high level actors’, described those who are ‘leaders in their fields and have the capacity and resources to create new positive opportunities for advisory members’ (Halsall et al., 2021, p. 625). Whilst a second paper proposed that one possible outcome when young people are formally paid is that they develop a professional identity and through this contribute to change (Chauhan et al., 2022). Despite these two accounts the vast majority of papers contributing to this CMO did not provide evidence that the personal benefits to the young participants led to service improvement. Moreover, one paper suggested a rival theory that in fact participating in co‐production work can be re‐traumatising for participants ‘(b)ecause our services can be quite stressful to work in’ and thus prevent or reverse psychological recovery (Oldknow et al., 2014, p. 20).

Further investigation into the contexts in which these reported gains for individual YEBE lead to the outcome of service improvement is therefore warranted.

Theory 3 transparency → respected/assimilate.

Theory 3 relates to the impact of being transparent with YEBE about the limitations for change within a mental health service. The limiting structural or institutional systems described in three papers contributing to this CMO included barriers from organisational bureaucracy (L. Hopkins, Foster, & Nikitin, 2018) and unspecified structural limitations to the change that is achievable (Mehrotra et al., 2017; Stubbs & Durcan, 2017). This theory was also supported by three papers highlighting that a lack of transparency in the form of misplaced encouragement of YEBE to set unrealistic expectations (described by Coates and Howe (2016, p. 296) as YEBE being told to ‘dream big’) ultimately leads to disappointment, frustration and conflict when their goals are not achieved. All six papers (see Table 7) therefore supported the theory that transparency around the limits of a programme is recommended for successful (if limited) co‐production in mental health services with young people.

Contrary to this, a rival theory was suggested in one paper that such compromise between fidelity to co‐production principles and pragmatic action can mean the co‐production fails to offer new perspectives as YEBE compromise their aspirations for change and assimilate: ‘the risk remains that youth advisors, through repeated exposure to what is possible within health services research and delivery, are over time acculturated to think exactly like the organisation, thus negating the original intent of engaging them’ (Canas et al., 2021, p. 1623). Thus, suggesting that co‐production which is transparent about the limitations cannot work in the sense of achieving transformative change in services due to institutional risk aversion.

The weight of evidence in this case appears to support being transparent about and working within structural limits. However, looking to substantive theory there is support for the rival theory that representation initiatives such as co‐production cannot achieve transformative change if project scope is limited from the outset. Another way to understand representation initiatives (like co‐production/youth participation) is as interconnected to identity politics as it is the participants youth and lived experience that legitimate their involvement. Táíwò (2022, p. 6) addresses these two positions in his book Elite Capture in which he highlights a tension between explanations which see identity politics as a threat to the established order and, conversely, explanations which say it is a tool ‘used by the bourgeoisie to maintain its class domination’. The latter is seen in practical terms as either when identity politics is symbolically performed to ‘pacify protestors without enacting material reforms’ or where existing institutions are rebranded via elements of identity politics, rather than being altered or replaced (Táíwò, 2022, p. 5). Co‐production ostensibly aims for the former (changing the established order) but is often accused of the latter (symbolism rather than material change).

Further investigation is therefore needed as to whether the, limited, service improvements achieved through transparency about structural/institutional limitations result in sufficient material change to services according to service users and YEBE co‐producers, or only symbolic change.

Theory 4 Shared power and responsibility → trusting relationships.

Theory 4 relates to the impact of sharing power and responsibility between stakeholders. The consensus was strong for this theory, with 20 of the included papers contributing to the CMO (see Table 8). Across the papers there was agreement that action can be taken to address power imbalances and share responsibility. Examples included acknowledging power differentials (Crofts et al., 2017), agreeing shared goals at the outset (Crofts et al., 2017; El Guenuni et al., 2022; Simmons et al., 2019) and ensuring YEBE outnumber staff (Allan & Travers‐Hill, 2019). The CMO is also supported by papers describing that where power and responsibility is not shared (i.e. services which are paternalistic, coercive or staff are untrained in collaboration) YEBE feel intimidated and isolated (Boswell et al., 2021) or lack a shared understanding (Ramey & Rose‐Krasnor, 2014), through this the status quo of staff making decisions is maintained (Allan & Travers‐Hill, 2019; O'Reilly et al., 2021) and consequently the service is not more responsive to needs of service users.

Looking to substantive theory this CMO is supported by partnership synergy theory (Lasker et al., 2001) that suggests that when different groups work together they can achieve more than those same groups would accomplish working separately. For co‐production in youth mental health this is the idea that the knowledge and experience of all stakeholders is utilised resulting in a ‘best of both’ scenario in which all parties influence the outcomes and there is a mutual boost in social capital.

No rival theories emerged from the literature. However, similarly to theory 1, the majority of the papers presented this as theory without concrete examples from the real‐world co‐production work they described. This CMO was presented as how co‐production is expected to work – but this requires further testing to understand if those co‐producing on the ground experience the theorised mutual respect and through this successfully contribute to service improvement.

Theory 5 Resources and flexibility → representation.

Theory 5 relates to the importance of having sufficient resources and being flexible to new ways of working. The importance of having sufficient resources, and flexibility to new ways of working was highlighted in 11 of the papers included in the review. Examples of resources discussed were money (to pay young people, transport costs or for materials) (Halsall et al., 2021; Oldknow et al., 2014) and mentorship provision (Chartier et al., 2022; Oldknow et al., 2014). Examples of flexiblity to new ways of working included using quota recruitment (Halsall et al., 2021), providing training (Swanton et al., 2007), holding meetings online (Brooks et al., 2021), changing the times of meetings (McCarty et al., 2022), providing support for the application process (Simmons et al., 2019), creating an informal environment (Young et al., 2023) , slowing the working processes to meaningfully listen (Monson & Thurley, 2011) and providing support with holistic needs (such as: housing, benefits applications, applying for passports/bank accounts, CV writing, job applications or support and advocacy during contact with the justice system) (Durcan et al., 2017).

*This theory is also* *supported* *by papers which highlighted that contextual factors such as s*ystemic racism within the mental health system (Le et al., 2022) and limited public awareness of participation practices (Brooks et al., 2021; Halsall et al., 2021) interact with insufficient resources and inflexibility to new ways of working to mean that YEBE who best represent the target group often lack information or encouragement to participate. The outcome in these scenarios was suggested to be that the co‐producers do not represent the target group for the service and only ‘safe’ ‘risk‐free’ and ‘effective’ narratives and ideas for change emerge (Kaiser et al., 2020, p. 115). Examples of this included poor advertising/outreach (Brooks et al., 2021; Halsall et al., 2021), cherry‐picking of narratives/participants (Kaiser et al., 2020; McCarty et al., 2022), and participants ageing but not moving on (Brooks et al., 2021). Minority groups which were highlighted as being excluded in the literature included young people from disadvantaged backgrounds (Howe et al., 2011), first nations young people (Chartier et al., 2022), those whose literacy has been affected by mental illness causing time out of education (Lindstrom et al., 2021) and those who have other commitments which clash with the timing of meetings (McCarty et al., 2022). Therefore, when resources are insufficient and working practices are inflexible the knowledge of YEBE from minoritized groups is not shared, as they are frequently not present.

The causal link between the context within this CMO (*YEBE from minoritized communities best understand their needs) and the outcome (of more culturally appropriate* *support* *) is* *supported* *by the* substantive theory of intergroup contact theory. This middle‐range theory suggests that when members of different groups come into contact with one another this can work to reduce prejudice and conflict between these groups (Brown & Hewstone, 2005). No rival theories emerged relating to this CMO, leading to a clear recommendation for co‐production to be sufficiently resourced and services to adapt working practices to be maximally inclusive in order that the co‐production participants best represent the target group for the mental health service. Theory 5 suggests that marginalisation in mental health services for youth is likely to increase when resources are scarce. It is therefore crucial to consider this in relation to the austerity agenda which has been seen across many health services and health service policy globally.

### Principal findings

The research question, the five identified mechanisms, and resulting final programme theory are shown in Table 10.

**TABLE 10:** Final programme theory.

Research question	For whom and in what circumstances does co‐production work in youth mental health services?	
Key generative mechanisms	A supportive organisational culture enabling YEBE to be perceived as credible knowers. YEBE authentically participating as co‐producers and benefiting from personal development (vocational skills, psychological wellness and new knowledge). Transparency about limits to the scope for change producing trust and shared understanding. Wider stakeholders sharing power and responsibility with YEBE leading to mutual respect, trust and stakeholders listening to one another. Programmes having sufficient resources and being flexible to new ways of working enhances accessibility for participation from minoritized groups.	
*Final Programme Theory*	Youth experts by experience (YEBE), particularly those from minoritised communities, provided with a supportive organisational culture can experience authentic engagement where their knowledge is perceived as credible by wider stakeholders. This leads to personal development for participating YEBE as well as service improvements from their input.	(Whom)
		*(in what circumstances)*
		*(co‐production works)*

## Discussion

Data from 57 papers contributed to five CMO configurations to describe the mechanisms by which co‐production in youth mental health services are linked to outcomes and influenced by context. In answer to the research question (see Table 10) these five CMOs contributed to the final programme theory:

Youth experts by experience (YEBE), particularly those from minoritised communities, provided with a supportive organisational culture can experience authentic engagement where their knowledge is perceived as credible by wider stakeholders. This leads to personal development for participating YEBE as well as service improvements from their input.

### Strengths, limitations and future research directions

Employing a realist approach to this review facilitated inclusion of rich data from a heterogenous sample of papers which would not have qualified for inclusion in a traditional systematic review. One weakness was the time limitation for the project, though this was mitigated through use of a youth reference group and expert panel (which included a second youth advisory group) as this enabled focussed research on the areas which are important to service users and other stakeholders.

Only two of the included papers were published in the global south ‐ Indonesia and India (Brooks et al., 2021; Mehrotra et al., 2017). Three possible explanations for this are available. Firstly, limiting the review to studies published in English limited the literature which could be included from the global south. Secondly, despite efforts during the searching and screening process to broaden terminology to include terms used for engagement work globally, few studies were found through the search. Thirdly, available studies from outside the global north which were screened at abstract or full‐text level often did not include youth engagement work that was carried through to implementation. The scarcity of studies from the global south in which youth engagement work is carried out through to implementation could be explained because of broader systemic barriers to this work taking place (for example due to reduced funding from governments and local authorities), or simply that though it is perhaps taking place in practice it has not been the focus of research in these contexts (influenced by where research funding is allocated). To bridge this knowledge gap it is essential that the mental health services, researchers and those funding youth engagement initiatives provide frameworks and resources that enable approaches such as co‐production within organisations to continue the move away from tokenism, and for research funding to be allocated to review progress in this area.

The skew towards studies from the global north is a significant limitation for the emerging theory which may therefore only be applicable to these cultural and economic contexts. To build a truly international overview the field would benefit from a future literature review which is not limited in scope to only resources published in the English language, such reviews are therefore a key priority for global health organisations to fund. There is also a possibility of a bias in our findings towards more positive depictions of engagement work reflecting publication bias of co‐production success stories.

The findings from this review will form the basis for a Delphi study with a range of expert stakeholders to consider the theory and it will be further refined, confirmed, or refuted. This process will involve comparison with the initial program theory developed with stakeholders to identify gaps and any further rival theories as well as comparison with published guidelines for co‐production work in youth mental health services. Finally, areas which still offer ambiguity will be tested in the field in an action research project within a mental health service for young people. The focus of this final stage will be directed by the Delphi results and may explore one/some of the areas identified as requiring further research, such as:

- Whether and how personal gains for participants leads to structural change.
- If transparency about the limitations of co‐production is helpful, or if it reveals the impotence of such programmes to challenge the status quo.
- Testing practical recommendations relating to creating an organisational culture of support (how to ensure sufficient resourcing, share power and work flexibly to maximise inclusion, representation and impact).

### Comparison with existing literature

Few examples were found describing co‐production in the strategic, or monitoring and evaluation domains. This reflects the findings of Gyamfi et al. (2007, p. 390) who saw in a national evaluation of youth involvement in mental health services, that involvement primarily occurred through advisory boards whilst ‘youth involvement in systems of care was found to be limited’. The only previous review published specifically on this subject is a 2021 systematic review which included just two papers, both scoring low for quality (Norton, 2021). Nevertheless, in support of theory 2, Norton (2021, p. 10) also concluded that ‘engaging in the process of co‐production, particularly within child and adolescent mental health, can have positive impacts on both an individual's mental health and sense of self’ and similar to theories 4 and 5 that effective co‐production requires redistributing power. A larger review published in 2023, but concerned with co‐production of youth mental health research rather than services, also had a congruent finding (McCabe et al., 2023). This review found support for using a flexible approach to youth engagement rather than always aiming for the gold‐standards of either partnership or youth‐led work. A realist approach has not been attempted in previous reviews on this subject.

### Dissemination of findings

The products of this review are suitable for guiding policy on co‐production in the sector and relevant to stakeholders in co‐production in youth mental health. To widen the potential audience,in addition to publication in a journal, findings will be available as a plain English summary including recommendations, devised with the youth reference group. To reach the broadest possible audience the expert panel and youth reference group will be encouraged to share outputs in their existing networks. The review findings and recommendations will shape the subsequent data collection stages of a more comprehensive PhD research project.

## CONCLUSION

The programme theory resulting from this review explains how co‐production is understood to work in mental health services for youth. The theory recommends building an organisational culture which supports co‐production with young people and views them as legitimate knowers (precisely because of their youth and lived experience) through sufficient resourcing, working flexibly, sharing power/responsibility and transparency regarding what is and is not feasible. One clear recommendation emerged for co‐production to be sufficiently resourced and services to adapt working practices to be maximally inclusive in order that the co‐production participants best represent the target group for the mental health service.

Nevertheless, three aspects are underdeveloped or untested and further information would be useful for understanding these missing links. Firstly, there was little evidence to support the claim that personal development for the YEBE participating leads to material organisational change. Secondly, it remains unconfirmed whether co‐production that is transparently ‘limited’ should always be aspired to or at times results in symbolism, assimilation, or unhelpful compromise rather than material change. Finally, practicable recommendations for how to build an organisational culture of support (how power and responsibility can be shared, what best constitutes flexible working practices and elements of sufficient resourcing) would be valuable information for those doing this work on the ground. These areas warrant further investigation.

## AUTHOR CONTRIBUTIONS

**Verity Rose Jones**: Conceptualization; data curation; formal analysis; funding acquisition; investigation; methodology; project administration; writing – original draft. **Justin Waring**: Supervision; writing – review & editing. **Nicola Wright**: Supervision; writing – review & editing. **Sarah‐Jane Fenton**: Methodology; project administration; supervision; writing – review & editing.

## CONFLICT OF INTEREST STATEMENT

The funders had no role in the design of the study, in the collection, analysis, or interpretation of data, in the writing of the manuscript, or in the decision to publish the results.

## ETHICS STATEMENT

N/A.

## PATIENT CONSENT STATEMENT

N/A.

## PERMISSION TO REPRODUCE MATERIAL FROM OTHER SOURCES

N/A.

## CLINICAL TRIAL REGISTRATION

N/A.

## Supporting information

Supporting Information S1

## Data Availability

Data are contained within the article.
