# Supplementary material for: A rapid realist review of literature examining Co‐production in mental health services for youth
Source: JCPP Adv. 2024 Aug 21;4(4):e12272. doi: 10.1002/jcv2.12272 (PMC11669775; doi:10.1002/jcv2.12272)
Supplement: Supplementary file 1 — Supporting Information S1 [file JCV2-4-e12272-s001.docx]

Supplementary Materials

| **Table S1**: Glossary of Terms | |
| --- | --- |
| Co-production | “Co-production is an equal relationship between people who use services and the people responsible for services. They work together, from design to delivery, sharing strategic decision-making about policies as well as decisions about the best ways to deliver services.” (Think Local Act Personal, 2021, p. 364) |
| Youth or young person | aged 10–25 (United Nations, 2023; WHO, 2021; World Economic Forum, 2020) |
| Mental Health Service | mental health care practiced within three areas: healthcare services (inpatient mental health and primary care/general hospitals), community mental health services, and beyond the health sector (e.g., schools, social services, and third sector organisations) (WHO, 2022a, 2022b) |
| Mental Healthcare | interventions aimed at improving mental health or wellbeing |
| Initial/Preliminary Theory | “An early theory informed by available evidence describing why, how and for whom the intervention is thought to work using a context-mechanism-outcome configuration” (Cooper et al., 2019, p. 2; Pawson & Tilley, 1997) |
| Refined Theory | “An initial theory that has been refined using primary or secondary evidence” (Cooper et al., 2019, p. 2; Pawson & Tilley, 1997) |
| Middle-Range-Theory | “theory that is not abstract to the point of being disconnected from the on-the-ground workings of programs, yet not so specific to pertain to one program” (Jagosh, 2019, p. 364) |
| Realism | the philosophical position that though objective reality is independent of us, it can only be understood through human interpretation and perception (sometimes described as sitting between positivism and constructivism) (Pawson et al., 2004; Searle, 1995). |
| Context-Mechanism-Outcome Configurations (CMOs) | “a heuristic used to generate causative explanations about outcomes in the observed data. A CMO configuration may be about the whole program or only to certain aspects… Configuring CMOs is a basis for generating and/ or refining the theory that becomes the final product of the review.” (Jagosh et al., 2015, p. 3) |
| Context | The backdrop of programmes: pre-existing conditions (in the physical/social environment) which influence the success or failure of different interventions or programmes (Cooper et al., 2019; Jagosh, 2019; Pawson & Tilley, 1997) |
| Generative Mechanism | Stakeholders’ response(s) to resources offered, the underpinning generative force that leads to outcomes (Cooper et al., 2019; Jagosh, 2019; Pawson & Tilley, 1997) |
| Outcome | Intended and unintended consequence of a mechanism operating within a context. The measurable impact at the behavioral, clinical, or system level (Cooper et al., 2019; Jagosh, 2019; Pawson & Tilley, 1997) |
| YEBE | “Youth Experts by Experience” (Acronym devised for this review) – young people with lived/living experience of mental distress, mental illness and/or mental health services |

| **Table S2:** Initial Programme Theory (if…then statements) | | | |
| --- | --- | --- | --- |
| Summary Statement | ‘if…then statement’ | | |
| SOME TOPICS ARE AVOIDED | If young people are only invited to co-produce on specific topics but others are avoided.  Then they may leave the role or give limited contributions.  Because they will not feel included as equals and will lack trust in the sincerity of the programme. | | |
| PROFESSIONAL’ SKILLS CAN BE OFFPUTTING | If ‘professional’ skills (like public speaking, and formal meetings) are needed to participate,  then the programme will lack accessibility and diversity.  Because a self-selecting group of stakeholders who find it accessible will participate and those who prefer other ways to contribute will drop out. | | |
| COMPULSORY TREATMENT | If young people’s mental health treatment is compulsory (e.g. ‘sectioning’)  then the co-production attempts in this service will fail.  Because, it will not be possible to have equal power among participants (which is needed for co-production). | | |
| CO-OPTATION / ASSIMILATION | If the co-production group do not have the power to make meaningful changes in the service,  then co-production only ‘works’ for those who support the status quo.  Because, contributors/co-producers change their expectations or compromise their principles to think ‘what can be done within the limits of this context?’ (instead of using outside-the-box thinking). | | |
| RIVAL THEORY: NON-ASSIMILATION | If the co-production group do not have the power to make meaningful changes in the service,  then service users leave the programme to find other ways to change the system/give up altogether.  Because the co-production programme is perceived by the users as tokenistic (and the service as self-congratulatory) | | |
| ‘AGE OUT’: LOSS OF ROLE | If there is an age restriction on participating,  then young people will experience a loss of role and identity  because when they ‘age out’ their knowledge will not be perceived as legitimate. | | |
| ‘AGE OUT’: PRODUCT LOSES RELEVANCE | If there is an age restriction on participating,  then the products of the co-production project will be perceived as less relevant over time  because the contributors ‘age out’ and their knowledge will be perceived as less legitimate. | | |
| SEEING RESULTS | If the project participants see results  then the ongoing co-production will be more successful  because trust is maintained | | |
| SHARED UNDERSTANDING OF CO-PRODUCTION | If training on co-production is provided to contributors/co-producers  then co-production ‘works’  because of a shared understanding which increases collaboration. | | |
| INCLUSIVE PRACTICE / CULTURAL COMPETENCE | If the facilitator uses inclusion practices and cultural competence  then marginalised groups are better included and more likely to stay involved (and the group is more ‘representative’ of the service users),  because participants experience greater psychological safety and feel welcome.  *(e.g. timing of meeting, flexible to meet online/in-person, mindful of language use, pronouns, trigger warnings, recruitment of programme participants, discussions of racism/discrimination)* | | |
| TIME, SPACE, MONEY, SNACKS | If the programme is well-resourced (funding, protected staff time, spaces to meet, snacks)  then co-production is successful in improving youth mental health services.  Because stakeholders feel valued and have the energy to engage and are able to put suggestions into practice. | | |
| COMMUNICATION BETWEEN MEETINGS | If there is a clear point of contact and good communication between meetings  then co-production is successful in improving youth mental health services.  Because, of a shared understanding and commitment to the work. | | |
| HONESTY ABOUT AIMS | If there are clear aims, transparency / honesty about the project scope/limitations and evaluation used,  then co-production is successful in improving youth mental health services.  Because, participants feel respected and have a shared understanding of success. | | |
| YP PRESENT FOR DECISION MAKING | If there young people are present when decisions are made  then co-production is successful in improving youth mental health services.  Because participants feel actually listened to | | |
| ASK: “IS CO-PRODUCTION RIGHT HERE?” | If sufficient planning takes place to make sure co-production is appropriate  then co-production is successful in improving youth mental health services.  Because, stakeholders ‘buy in’ to the usefulness of using co-production. | | |
| TALKING ABOUT POWER | If power is discussed and a process is in place for redressing hierarchies and power imbalance,  then power is shared more equally among participants (and the co-production more successful).  Because, participants are aware of their relative power and the shared agreement to re-balance this. | | |
| YOUTH-INVOLVED FROM THE START | If young people are involved from the outset of a co-production programme,  then the programme is more likely to truly reflect the needs and ideas of those with lived experience.  Because, young people shape the direction and priorities of the work, and identify the focus at the co-planning stages. | | |
| YOUTH-INVOLVED TO THE END | If young people are involved throughout (to the end of a programme)  then the programme is more likely to truly reflect the needs and ideas of those with lived experience.  Because, young people shape the direction and priorities of the work | | |
| RECOGNITION | If there is appropriate recognition of stakeholder contribution  then the programme has a positive psycho-social impact on participants (e.g. improved self-esteem/efficacy)  because stakeholders feel valued.  *(recognition includes: payment, named author, speaking at events, thanked publicly etc).* | | |
| SUPPORT AVAILABLE | If psychological or pastoral support is available for contributors/co-producers,  then the programme has a positive psycho-social impact on participants.  Because of increased psychological safety and reduced psychological harm. | | |
| AGE LIMIT GIVES LEGITIMACY | If there is an age restriction on participating,  then the programme retains legitimacy  because the stakeholders youth is perceived to give legitimacy to their knowledge. | | |
| *other factors that influence* | CONTEXTUAL FACTORS   - Age of young people - Personal factors influence participation - Literacy of stakeholders - Mental health literacy of stakeholders - Unspoken mental health lived-experience of staff - Co-production is or isn’t well-integrated into the service (e.g. invited to existing meetings) - Type of mental health service (who are the end users) - What is being co-produced   Social support is available for young people | MECHANISMS   - Mutual benefit - Shared purpose - Relationship building - resource: journeying together, response: equality of esteem / feeling respected - Who is targeted in recruitment to co-production programmes (different skillsets/diverse groups) - Programmes which compromise or take a pragmatic approach rather than aiming for ‘best practice’ co-production - Methods of co-production   Level or lack of support offered for the transition to ‘very different’ adult PPI/co-production work | OUTCOMES   - Finding agreement - between majority / minority views - Who gets included: Carers, teachers, support workers, family members etc also have relevant knowledge about youth mental health services - Balancing evidence based practice with expertise from experience |


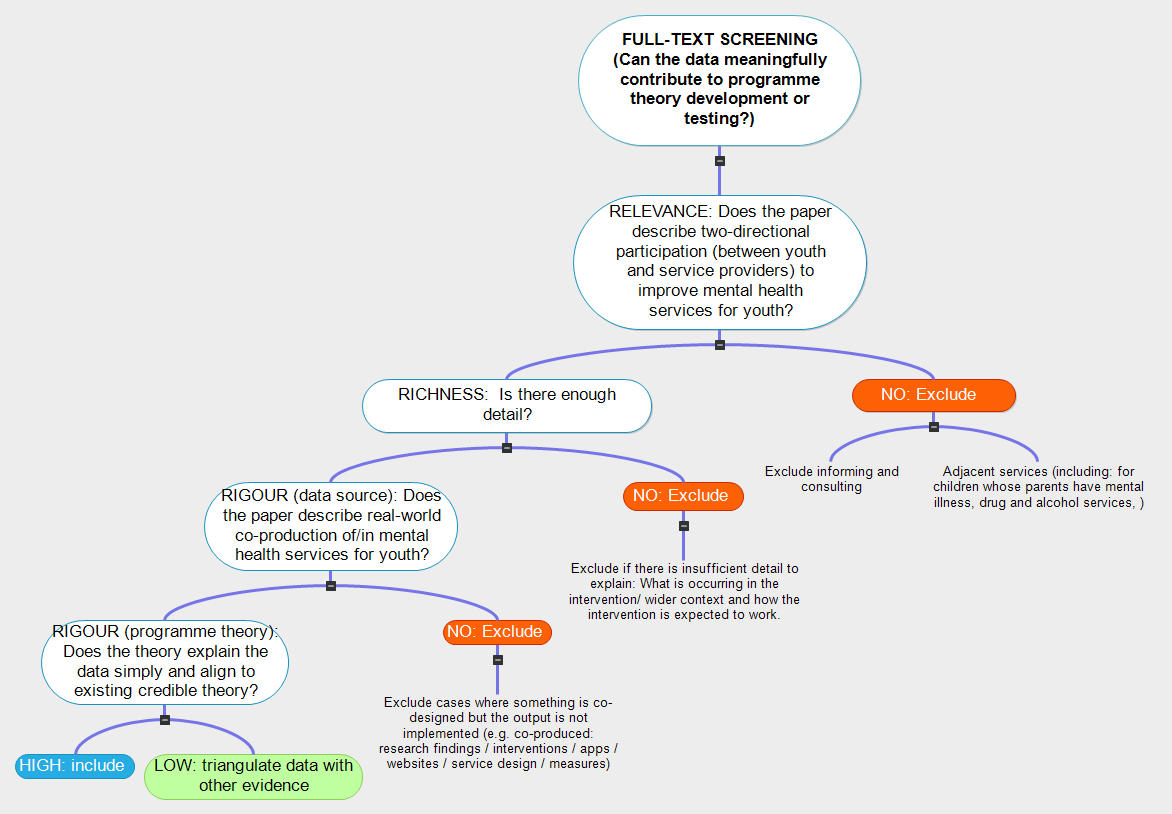


**Figure S1:** Relevance, Richness and Rigour Assessment Process During Full-Text Screening

| **Tables S3-S10:** Full Search Terms |
| --- |
| [**Link to pdf**](https://bham-my.sharepoint.com/personal/vrj299_student_bham_ac_uk/Documents/LITERATURE%20REVIEW/REVIEW/JCCP%20Advances%20Submission/Full%20Search%20Terms.pdf) |

| **Table S11:** Study/Project Characteristics Data Extraction |
| --- |
| Author, Date |
| Research question / aims |
| Data collection method |
| Data analysis method |
| Type of mental health service |
| Study / report participants |
| Country/Region |
| Mental health issue (if specified) |
| Project name |
| What is being co-designed/co-produced |
| Co-production theoretical framework /model |
| Co-production activities (approach /method /process) |
| Any definition of co-production used? |
| Co-production prog. participants |
| Renumeration / recognition |
| Marginalised group/ protected characteristics |

| **Table S12:** Examples of CMO Data Extraction | | | | | |
| --- | --- | --- | --- | --- | --- |
| **Author, Date** | **Causal summary** | **CONTEXT** | **MECHANISM - RESOURCE (if) how,why,whom** | **MECHANISM - RESPONSE/REASONING (because)** | **OUTCOME (then)** |
| 42.Mehrotra et al, 2017 | young people are given information on what is/isn't feasible > use this knowledge to plan (and implementation of plans more likely) | South India - pilot programme was open to any ideas and little/no implementation happened – YP got stuck at planning phase | limits / what is feasible is provided for young people at the outset - they then select/modify existing plans | Young people can select/modify ideas | concrete plans emerge and are implemented |
| supporting data |  |  | "different kinds of feasible activities as well as related resource materials is mailed to the members to ease their burden in term of searching for resources /developing material for use." | The youth have the freedom to select/and modify any of the ideas provided. | support offered for coming up with concrete plans was increased by providing resources (detailing feasible potential activities) and materials to carry out activities. |
| 28.Hopkins, Foster and Nikitin, 2018 | compromise fidelity and pragmatism > still felt to be meaningful (and allowed getting things going) | not possible for all course facilitators to be young people | compromise between fidelity to the model and pragmatic action | felt as intellectual tension/ philosophical differences but also compromise is still meaningful to youth | getting things going |
| supporting data |  | We didn’t know at the beginning if all of the people who facilitated the courses needed to be young people. We realised quite early on that that was probably going to be impossible for a lot of different reasons. | "compromises required between fidelity to the Discovery College model and the pragmatics of establishing action." "absolute fidelity to the model is not always possible, nor indeed, essential or even desirable" "“Perhaps the biggest new learning (and highly significant in the field of youth-focused Recovery Colleges) is that whilst there is value in having a portion of the peer/lived experience workforce that is “youth” in age, the reality is that that may not be feasible at the outset. Having a workforce of people who can draw on a lived experience in a way that is meaningful to youth, however, is both possible and appropriate.”" | intellectual tensions / philosophical differences / compromise |  |
| 54.Stubbs & Durcan, 2017 |  |  | boundaried, and therefore authentic co-production on specific projects expertise of YP and clinical staff is recognised | young people feel valued and empowered | YP saw themselves differently (+ treat like a man, act like a man) |
| supporting data |  | reach its most marginalised communities | young people and staff work together on discrete and structured pieces of work, e.g. planning a trip, designing a schools programme. Project Future works on the basis that coproducing a project or piece of work requires scaffolding (attuning to an individual’s developmental needs and providing the necessary support for them to achieve their goal) (Wood, 1976 in Stubbs and Duncan, 2017) for it to be helpful, genuine, achievable and wellbeing-enhancing… It would be a mistake to see coproduction as everyone having an equal and shared role, and the clinical expertise is core to the project’s effectiveness. | Project Future was perceived to be an environment that made young people feel safe, respected, accepted, provided with opportunities, empowered, special, supported and listened to. "young people feel valued and empowered" | This enabled young people to see themselves in different ways, access new opportunities, and envision and work towards a “future self”. "“If you’re treated like a little boy, you’ll act like a little boy. If you’re treated like an animal you’ll act like an animal. Here you’re treated like a man, so you act like a man…”" |
| 6.Canas et al., 2021 | transparency regarding limits to potential change > assimilation of YP | institutional risk aversion | there is transparency /authenticity about what is achievable | Youth’s enthusiasm for change shifts and YP begin to think exactly like staff (socialisation into the organisation) | negates purpose of engaging them to think differently - the co-production work fails to offer new perspectives/meaningful change |
| supporting data |  | p. 1623 "institutionalized risk aversion of health services organizations" p.1622/3 "professional expectations of what it means to interact with youth” | p.1623 “repeated exposure to what is possible”  p. 1621 "The service providers continually recruited new youth advisors, restarting processes of building trust and familiarising them to the organisational language and culture" p.1622 | p. 1623 "the risk remains that youth advisors, through repeated exposure to what is possible within health services research and delivery, are over time acculturated to think exactly like the organisation, thus negating the original intent of engaging them" | p. 1622 "There is a misalignment between ideals of youth engagement (/fervour for things to be youth-led) and their realization in practice in a service delivery setting" |

**
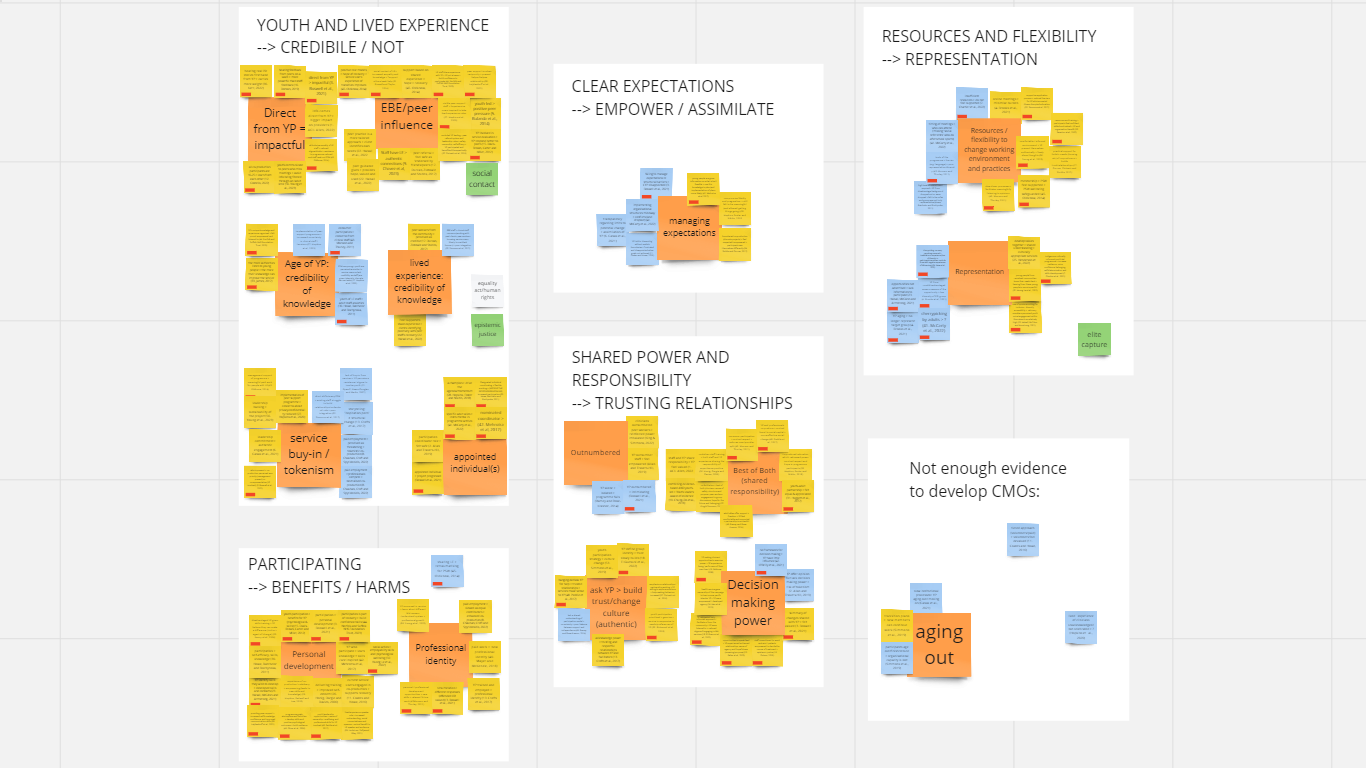
**

**Figure S2:** Grouping by Demi-Regularities [**link to Miro board**](https://miro.com/welcomeonboard/YWFmY01rU3JsOWR1QktnYmdLdjZaVTVMTXBmdDF6RnhZQWVaWXZDSzNKdng0bkhsT2RYbW1EOWxMQ1hLMGR4cHwzNDU4NzY0NTcyOTM3NzA5NTY5fDI=?share_link_id=910053437793)

| **Table S13:**  Contexts, Mechanisms and Outcomes by Theme | | | | | | | |
| --- | --- | --- | --- | --- | --- | --- | --- |
| **Keyword** | **Works?** | **CONTEXT** | **MECHANISM - RESOURCE (if) how,why,whom** | **MECHANISM - RESPONSE/REASONING (because)** | **OUTCOME (then)** | **Middle range theory?** | **Papers** |
| YOUTH AND LIVED EXPERIENCE --> CREDIBLE /NOT | What works? | staff committed to co-production YP | Experts by experience's youth and lived experience | YP share LE directly with staff / service users and their knowledge is seen as credible (unique knowledge due to youth). Staff authentically engage with YP (allies, leadership presence), Co-production is felt to be a priority, YP feel safe. YP's knowledge is listened to. They feel listened to and empowered. (Coming directly from YP with LE the information is more powerful/impactful, provides hope of recovery (SU)/ empathy (staff) and instils safety/ease for service users.) | Young people's knowledge is used to improve the service. (The advice provided is valued and used (by staff and service users), service users are more easily recruited to and remain engaged in treatment, they 'respond better' to peers and it avoids a helper/helpee relationship). Project has momentum and is sustainable | epistemic injustice, social contact theory, Luke's three dimensions of power | 1, 2, 3, 5, 6, 9, 12, 1,5 16, 17, 22, 27, 28, 30, 32, 36, 38, 41, 42, 44, 45, 51, 56 |
| YOUTH AND LIVED EXPERIENCE --> CREDIBLE / NOT | What doesn't work? | Organisational culture: staff resistant to co-production with YP (systems around the project limit the scope) | Experts by experience's youth and lived experience | Seen as not-credible (because of youth), Staff are anxious and concerned, YP perceive resistance and feel unsupported. Staff compete with YP and/or limit their involvement | Input is tokenistic/restrained and cannot shape real change or implementation fails ("the co-production work fails to offer new perspectives/meaningful change"). Young people are given narrow, discrete tasks and impact is limited | epistemic injustice, Luke's three dimensions of power | 8, 13, 21, 27, 30, 43, 52 |
| PARTICIPATING --> BENEFITS / HARMS | What works? | Where CYP are co-producing mental health services | YP participate in the meaningful activity of co-production | improved self-esteem/efficacy, confidence, resilience, feel empowered, social connectedness, build professional identity (skills of public speaking, employability, communication, time management, teamwork) and develop knowledge (of the system, research, evaluation, mental health conditions) | Personal development of CYP: psychological recovery, professional skills and knowledge (suggested that YP can use these new skills/confidence to make change) | Occupational therapy: meaningful activity engagement is therapeutic (+ candidacy theory) | 3, 8, 11, 13, 15, 23, 26, 29, 30, 32, 35, 38, 39, 40, 42, 43, 44, 46, 48, 56 |
| PARTICIPATING --> BENEFITS / HARMS | What doesn't work? |  | sharing lived experiences of mental illness | negative impact on PSW (re-traumatising) | peer support workers become unwell through doing the role |  | 45 |
| CLEAR EXPECTATIONS --> EMPOWER / ASSIMILATE | What works? | systems around the project limit the scope | Transparency of limitations (e.g. structural)/ what is and is not feasible (incl. compromise between fidelity to the model and pragmatic action) | Young people use this knowledge to plan, feel respected and empowered (authentic) or YP compromise on their aspirations for change and assimilate ("YP begin to think exactly like staff") | concrete plans emerge and implementation of the plans is more likely |  | 28, 42, 54 (6 - assimilate) |
| CLEAR EXPECTATIONS --> EMPOWER / ASSIMILATE | What doesn't work? | organisational bureaucracy and prejudices/concerns of staff/institutional risk aversion | Service is not transparent about limitations - YP are encouraged to 'dream big' | disappointment, frustration and conflict when goals are not achieved | goals are not achieved, YP drop out, negative impact on participants |  | 3, 11, 41 |
| SHARED POWER / RESPONSIBILITY --> TRUSTING RELATIONSHIPS | What works? | status quo - staff make decisions / inevitable unequal power between adults and youth. Strengths and assets of the community of YP. | Genuine sharing of decision-making power and responsibility including listening to YP | Experienced as positive for both staff and YP. Trustful and respectful relationships with mutual respect and agency, in which YP feel valued, hopeful, appreciated, supported, comfortable, empowered and at times equal. | knowledge and experience of both groups is used, services are more responsive to the needs and preferences of YP. Mutual boost to social capital and programme better for having both stakeholder groups involved. | partnership synergy theory | 1, 2, 3, 6, 10, 13, 16, 18, 19, 20, 24, 26, 28, 31, 37, 43, 48, 49, 50, 53, 57 |
| SHARED POWER / RESPONSIBILITY --> TRUSTING RELATIONSHIPS | What doesn't work? | paternalism, coercive institutional environments, negative stereotyping of youth, staff untrained in co-creation/collaboration. cultural gap between youth engagement and the organizational climate typical of child and youth mental health services. | Young people lack decision making power | uncertainty | tokenism, little influence, poor balance between support and independence |  | 2, 3, 47, 49 |
| RESOURCES AND FLEXIBILITY --> REPRESENTATION | What works? | participants wish to help others with their stories. young people from minoritized communities know their needs best | flexibility and resources (resources, training, meetings online, supportive application process, informal environment, mentorship, slowing working processes to meaningfully listen and support with holistic needs (e.g. Housing benefits applications, applying for passports and bank accounts, CV writing, job applications or support and advocacy during their contact with the justice system) | participants feel supported, confident, skilled, valued, comfortable and relationships build | YP from minoritized communities are included and listened to (e.g. using quota recruitment). YP's opinions are heard, burden to participate is reduced so the programme is more inclusive, YP with disrupted education are not excluded, YP are able to present themselves authentically and freely share thoughts. group is representative of target population and mental health programmes offer better support through culturally appropriate services | epistemic injustice and social contact theory | 4, 7, 17, 23, 25, 32, 43, 45, 52, 55, 56 |
| RESOURCES AND FLEXIBILITY --> REPRESENTATION | What doesn't work? | limited 'societal understanding' / public awareness of PPI, systemic racism within the mental health system | insufficient resources, inappropriate programme tools/techniques (e.g. jargon), reliance on YP volunteering | YP who represent target group lack information or encouragement to participate. participants do not feel supported, lack shared understanding (e.g. jargon used), feel devalued if unpaid in tiered model | unrepresentative group participate (due poor advertising/outreach, cherry picking of narratives/participants, YP aging). poor representation / lack of diversity (stakeholders cannot participate/ fully participate) (e.g. inpatients, first nations YP, 'social referents' in sports teams with timetable clash. lack of diversity in participants/narratives, does not represent target group (only 'safe' 'risk-free' and 'effective' narratives emerge. | epistemic injustice | 4, 7, 23, 34, 41, 43 |

| **Table S14:** Theory Refinement |
| --- |
| [**Link to pdf**](https://bham-my.sharepoint.com/personal/vrj299_student_bham_ac_uk/Documents/LITERATURE%20REVIEW/REVIEW/Supplementary%20material/Theory%20Refinement.pdf) |


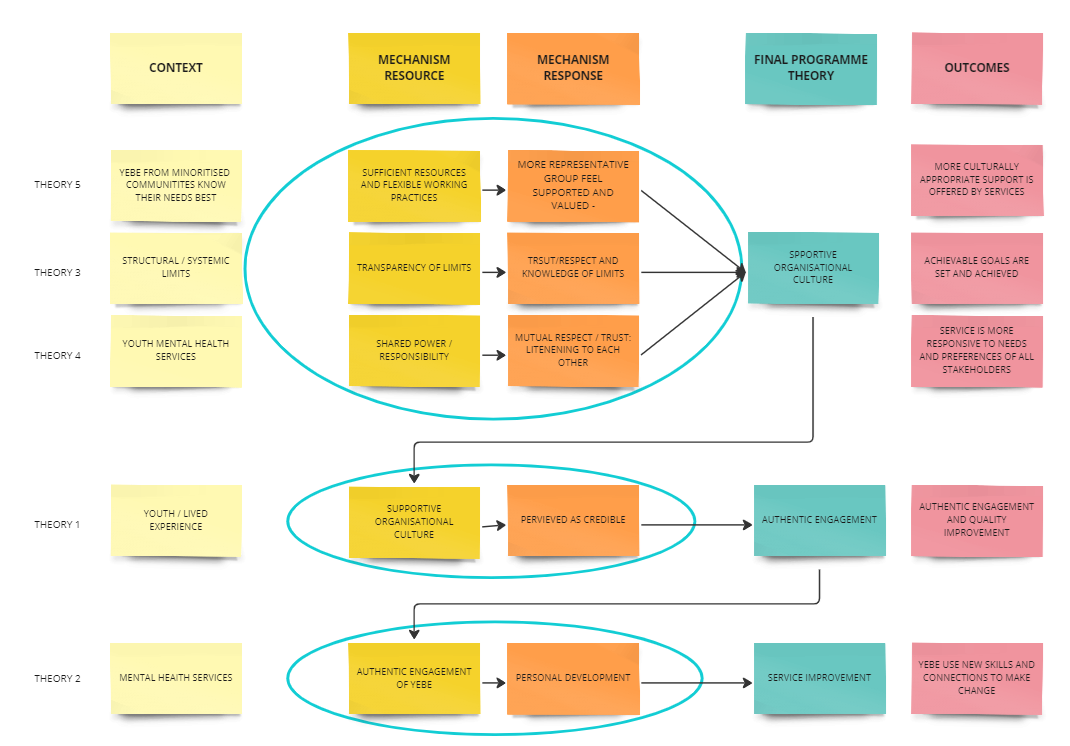


**Figure S3:** Conceptual Map of Full Programme Theory

**Figure S4:** Methods of Co-production

**Figure S5:** What is Being Co-produced

| **Table S15:** Full Extraction Spreadsheets |
| --- |
| [**Link to table**](https://bham-my.sharepoint.com/personal/vrj299_student_bham_ac_uk/_layouts/15/guestaccess.aspx?share=EZK5-xStB5JOvyJzZabRMB8BwpI_hOh_CG-mVzWVGJCXPA&e=SCEelD) |

| **Table S16:** Summary of Included Papers |
| --- |
| [**Link to table**](https://bham-my.sharepoint.com/personal/vrj299_student_bham_ac_uk/Documents/LITERATURE%20REVIEW/REVIEW/Tables%20and%20graphics/Table%20S15%20Summary%20of%20Included%20Papers.xlsx) |
